# Notable dysthymia: evolving trends of major depressive disorders and dysthymia in China from 1990 to 2019, and projections until 2030

**DOI:** 10.1186/s12889-024-18943-7

**Published:** 2024-06-13

**Authors:** Wei Wang, Yihe Wang, Feng Wang, Hui Chen, Xiaqing Qin, Lexia Yang, Xiaorong Yang, Lejin Yang

**Affiliations:** 1 Department of Psychology, Qilu Hospital of Shandong University, 107 Wenhuaxi Road, Jinan, Shandong 250012 China; 2 Department of Neurology, The Second Hospital of Shandong University, Jinan, China; 3 Department of Education, Guangxi Normal University, Guilin, China; 4 Clinical Epidemiology Unit, Qilu Hospital of Shandong University, Jinan, China; 5 Clinical Research Center of Shandong University, Qilu Hospital, Cheeloo College of Medicine, Shandong University, Jinan, China; 6 Department of Psychology, Shandong Normal University, Jinan, China; 7 Nursing Department, The Third Hospital of Jinan, Jinan, Shandong 250000 China

**Keywords:** Dysthymia, Major depressive disorders, Disability-adjusted life years, Temporal trend, Risk factors

## Abstract

**Background:**

Depressive disorders have been identified as a significant contributor to non-fatal health loss in China. Among the various subtypes of depressive disorders, dysthymia is gaining attention due to its similarity in clinical severity and disability to major depressive disorders (MDD). However, national epidemiological data on the burden of disease and risk factors of MDD and dysthymia in China are scarce.

**Methods:**

This study aimed to evaluate and compare the incidence, prevalence, and disability-adjusted life-years (DALYs) caused by MDD and dysthymia in China between 1990 and 2019. The temporal trends of the depressive disorder burden were evaluated using the average annual percentage change. The comparative risk assessment framework was used to estimate the proportion of DALYs attributed to risk factors, and a Bayesian age-period-cohort model was applied to project the burden of depressive disorders.

**Results:**

From 1990 to 2019, the overall age-standardized estimates of dysthymia in China remained stable, while MDD showed a decreasing trend. Since 2006, the raw prevalence of dysthymia exceeded that of MDD for the first time, and increased alternately with MDD in recent years. Moreover, while the prevalence and burden of MDD decreased in younger age groups, it increased in the aged population. In contrast, the prevalence and burden of dysthymia remained stable across different ages. In females, 11.34% of the DALYs attributable to depressive disorders in 2019 in China were caused by intimate partner violence, which has increasingly become prominent among older women. From 2020 to 2030, the age-standardized incidence, prevalence, and DALYs of dysthymia in China are projected to remain stable, while MDD is expected to continue declining.

**Conclusions:**

To reduce the burden of depressive disorders in China, more attention and targeted strategies are needed for dysthymia. It’s also urgent to control potential risk factors like intimate partner violence and develop intervention strategies for older women. These efforts are crucial for improving mental health outcomes in China.

**Supplementary Information:**

The online version contains supplementary material available at 10.1186/s12889-024-18943-7.

## Introduction

China, with 18% of the world’s population, has undergone significant economic and institutional changes over the past three decades. As such, it serves as an excellent research sample for other developing countries and the world as a whole. Despite its rapid economic development, China faces social issues like increasing competitive pressure, rising medical costs, widening poverty gaps, and increasing psychological stress, which have contributed to a rise in the incidence of depression over time [1, 2]. This trend has posed a serious threat to society and personal lives [3], necessitating the urgent need to understand the epidemic trend of depressive disorders and develop effective strategies to address them.

While major depressive disorder (MDD) is often the focus of attention due to its severity and short-term effects, another subtype of depression, dysthymia, is frequently overlooked [4, 5]. Dysthymia, defined by the ICD-10 as a persistent depressive mood lasting for more than two years and with lower severity than MDD, is associated with significantly impaired quality of life, despite its milder symptoms [6]. For instance, individuals with dysthymia exhibit lower rates of full-time employment and are more likely to depend on public assistance compared to the general population and individuals with MDD [7]. Moreover, dysthymia is characterized by higher prevalence in the general population, greater childhood adversity, more functional impairment, and a higher risk for suicide attempts [8]. Compared to the episodic form of MDD, dysthymia is associated with more severe life damage and higher social and economic costs [9]. Thus, analyzing the differences between MDD and dysthymia may yield more disease phenotypes for exploratory research on etiology and treatment [8]. However, comparative research data on MDD and dysthymia remain scarce [8].

Currently, the pathogenesis of depression remains unclear and its high recurrence rate continues to pose a significant treatment burden. Ren et al. reported that the prevalence and DALY rate of depression had increased to varying degrees across all age groups in China from 1990 to 2017 [2]. However, their study only analyzed the prevalence and DALYs of depression by age, gender, and province. Yueqin Huang et al. conducted a cross-sectional epidemiological survey of the prevalence of adult depression in 157 nationwide representative population-based disease surveillance points in 31 provinces in China [2]. The results showed a lifetime weighted prevalence of 3.4% for MDD and 1.4% for dysthymia. However, dysthymia is considered to be equally disabling and clinically severe as MDD. Therefore, the identification, prevention, and treatment of dysthymia should be considered as important as that of MDD. Alarmingly, only 9.5% of the 1007 participants with depressive disorders used mental health services and only 0.5% of those with depressive disorders received adequate treatment. Therefore, detailed epidemiological characteristics of MDD and dysthymia, especially risk factors, are important to improve treatment engagement and reduce the burden of disease [10].

However, few studies have thoroughly assessed, compared, and projected the burden of MDD and dysthymia, along with their risk factors, in China. Such studies could greatly assist in the formulation of policies for preventing and managing depressive disorders. For example, the significant increase in disability-adjusted life years(DALYs) of MDD attributable to bullying victimization over the last three decades requires extra attention [11]. Additionally, a possible causal relationship between bullying victimization and depressive disorders has been suggested [12]. Bullying victimization not only increases the risk of mental disorders but also carries significant direct costs for individuals and society as a whole [13]. Unfortunately, the burden of disease resulting from bullying victimization was not assessed until the Global Burden of Disease (GBD) 2017.

The aim of this study is to evaluate and compare the burden of MDD and dysthymia in China from 1990 to 2019, including temporal trends by age, sex, disease subtypes, and risk factors. Furthermore, this study seeks to project the burden of dysthymia and MDD separately from 2020 to 2030. This research can aid in the evaluation of current prevention strategies and provide additional theoretical support for their revision.

## Methods

### Depression definition and data sources

All data and analyses presented in this study were based on the GBD Study 2019, which provides estimates of incidence, prevalence, deaths, and DALYs in different countries and regions from 1990 to 2019. The GBD Study employs meticulous methods that have been described in detail in earlier publications [14, 15]. Using the Diagnostic and Statistical Manual IV (DSM-4) and the International Classification of Diseases 10th revision (ICD-10), the GBD Study examined two major categories of depression: MDD and dysthymia. The codes used to identify depressive disorders were DSM-IV-TR: 296.21-24, 296.31-34, 300.4 and ICD-10: F32.0-9, F33.0-9, F34.1, which covered MDD and dysthymia and excluded cases due to general medical conditions or substance use. To estimate the prevalence, incidence, duration, and excess fatality associated with depressive disorders, the GBD Study conducted a comprehensive search of PsycInfo, Embase, PubMed, the grey literature, and consulted with experts. A total of 517 and 107 original data sources were collected for MDD and dysthymia, respectively, to enable global assessment of depressive disorders. Detailed information on the search strategies, methodologies, and estimation of depression can be found on the GBD Study website (http://ghdx.healthdata.org/gbd-2019).

To analyze the burden of depressive disorders in China, we utilized the GBD database and selected China as the location, “depressive disorder” as the cause, and “incidence,” “prevalence,” and “DALYs” as measures. The DALYs were computed by adding disability-adjusted life years (YLDs) and YLLs years of life lost (YLLs) in GBD study. Because depressive disorders are nonfatal disease, the DALYs due to depressive disorders are equivalent to the YLDs, which were computed by sequela as prevalence multiplied by the disability weights (DW) for the health state associated with that sequela [14]. In calculating the 95% uncertainty intervals (UIs), we utilized the 25th and 975th ordered percentiles of 1,000 random draws in the GBD study. Age-standardized rates for the incidence, prevalence, and DALYs of depressive disorders were calculated using the World Health Organization (WHO) World Standard Population Distribution (2000–2025). Additionally, the United Nations World Population Prospects 2019 Revision was used to predict the population.

### Ethics approval and consent to participate

The authors confirm that this study was conducted in compliance with the ethical standards set by national and institutional committees on human experimentation, as well as the Helsinki Declaration of 1975 (revised in 2008). The study was approved by the Ethics Committee of Qilu Hospital of Shandong University (approval number KYLL-202,011(KS)-239). As the GBD 2019 study is a public database with all data being anonymous, no further ethical approval was required for this study.

### Data analysis

This study aimed to describe the shift in incidence, prevalence, and DALYs of depressive disorders in China from 1990 to 2019, with all cases divided into 5-year age groups. The average annual percentage change (AAPC) was calculated to quantify temporal trends of incidence, prevalence, and DALYs [16–18]. The regression line was fitted to the natural logarithm of the rates, i.e., y = α + βx + ɛ, where y = ln (rate) and x = calendar year, and the AAPC was calculated as 100 × (exp(β)-1). A 95% confidence interval (CI) of AAPC was also computed. To estimate the proportion of DALYs attributed to potential risk factors, the comparative risk assessment (CRA) framework and three well-established risk factors for depressive disorders (bullying victimization, child sexual violence, and intimate partner violence) estimated in the GBD 2019 study were applied in the present study [15]. According to the World Cancer Research Fund grades of convincing or probable evidence, intimate partner violence, bullying victimization and childhood sexual abuse were identified as attributable risk factors of MDD. The disease burden attributable to risk factors was estimated the following six key steps: inclusion of risk–outcome pairs in the analysis; estimation of relative risk as a function of exposure; estimation of exposure levels and distributions; determination of the counterfactual level of exposure, the level of exposure with minimum risk called the theoretical minimum risk exposure level (TMREL); computation of population attributable fractions and attributable burden; and estimation of mediation of different risk factors through other risk factors [19]. Bayesian age-period-cohort analysis with integrated nested Laplace approximation was used to project the numbers of cases, prevalence, and DALYs for depressive disorders by disease subtypes from 2020 to 2030. This method has been proven to have better accuracy in forecasting non-communicable diseases, especially in non-longer projection years, compared with other models [20, 21]. The Bayesian age-period-cohort model (The default parameters have been used) examined the multiplicative effect of age, period, and cohort: η_ij_ = log(λ_ij_) = µ + α_i_ + β_j_ + γ_k_. In this model, λ_ij_ stand for the number of cases, µ denotes the intercept, and α_i_, β_j_ and γ_k_ were age, period, and cohort effects, respectively. In addition, the absolute numbers of incidence, prevalence, and DALYs were calculated if the rates annually decreased or increased by 1% or remined stable based on data in 2019. Data were conducted with the R (Version 4.2.0), including the following packages: ggplot2, RColorBrewer, BAPC, and INLA.  

## Results

### Burden of depressive disorders consistently decreased during the past three decades in China

**Fig. 1:**
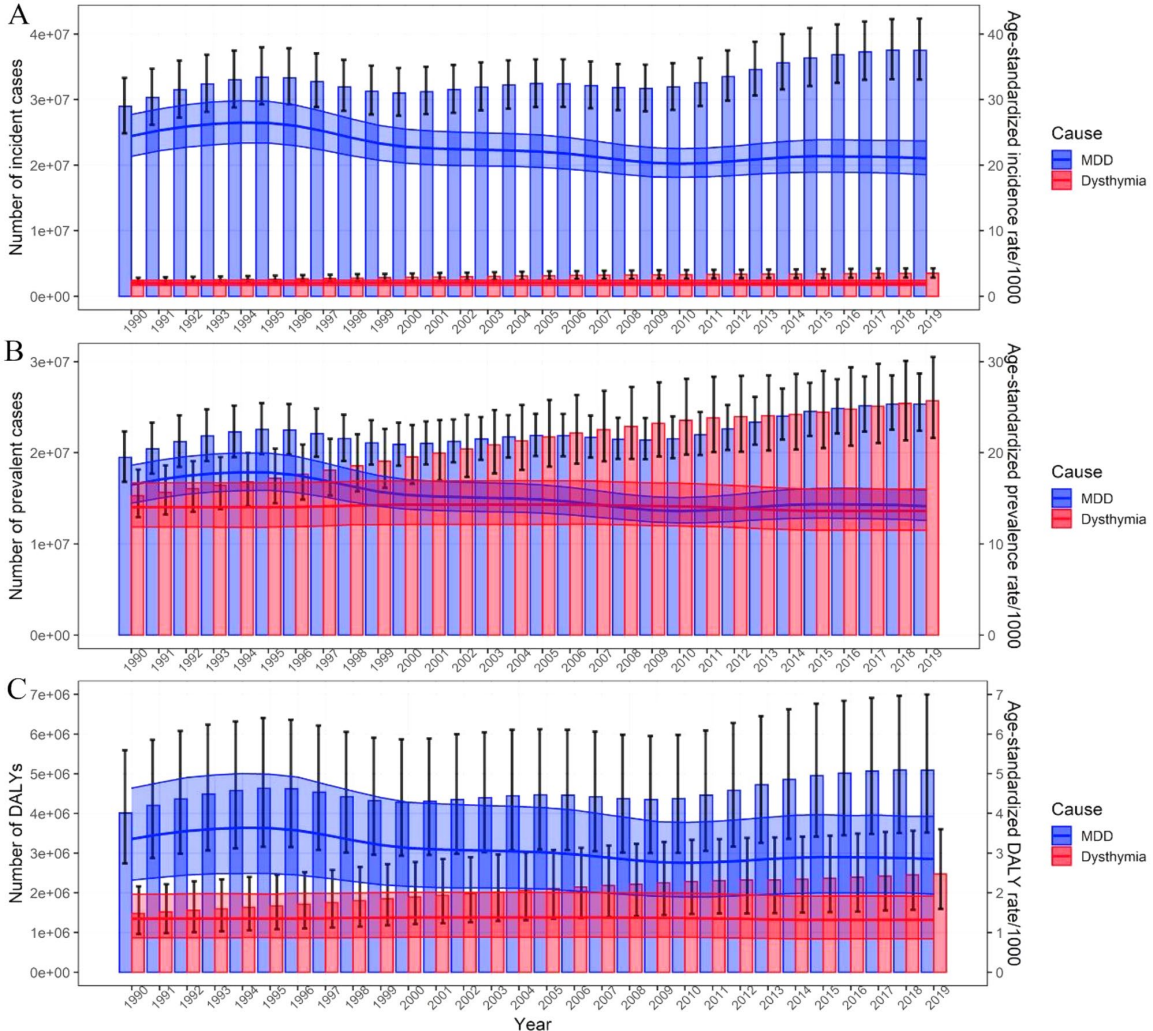
The incidence, prevalence, and DALYs of depressive disorders in China by categories and year. The numbers and age-standardized rates of incidence (**A**), prevalence (**B**), and DALYs (**C**) of depressive disorders by categories. Shading indicates the upper and lower limits of the 95% uncertainty intervals (95% UIs). DALYs, disability-adjusted life-year

**Fig. 2:**
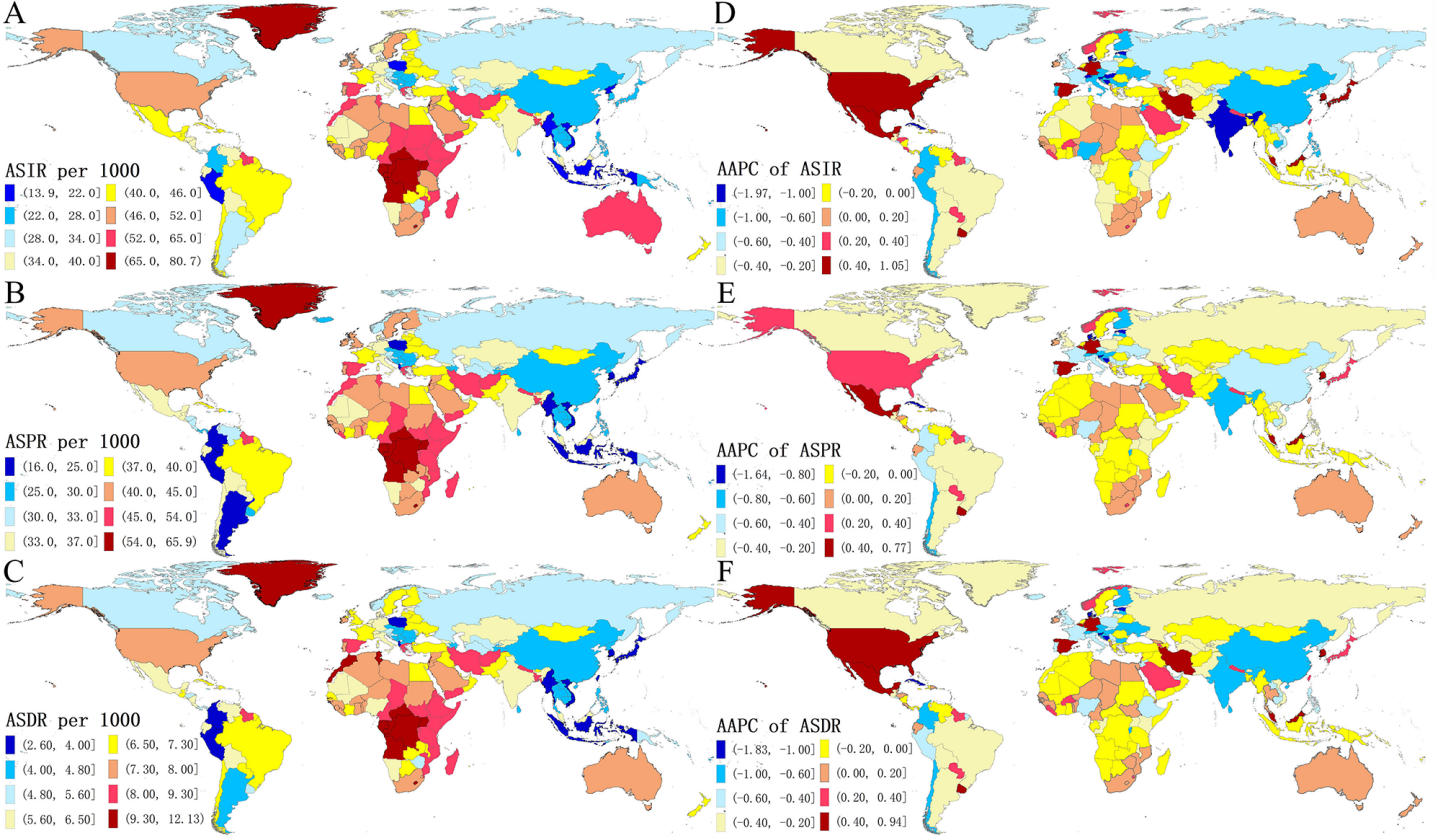
The global ASIR of depressive disorders in 204 countries and territories. The ASIR (**A**), ASPR (**B**) and ASDR (**C**) of depressive disorders in 2019; the AAPC of ASIR (**D**), ASPR (**E**) and ASDR (**F**) of depressive disorders from 1990 to 2019. ASIR, age-standardized incidence rate; ASPR, age-standardized incidence rate; ASDR, the age-standardized rate of DALYs rates; AAPC, Average annual percentage change

The age-standardized incidence rate (ASIR) of depressive disorders in China decreased over the past 30 years, with an AAPC of -0.82 (95% CI: -1.00, -0.65) (Table 1; Figs. 1 and 2). Furthermore, the changing trends in age-standardized prevalence rate (ASPR) also decreased with an AAPC of -0.53 (95% CI: -0.61, -0.45), as did the age-standardized DALY rate (ASDR) with an AAPC of -0.69 (95% CI: -0.81, -0.57) for depressive disorders (Table 1; Figs. 1 and 2). These indicators remained relatively stable when considering the growth in population numbers and the increasing aging population in China.

**Table 1:** The number of burden cases and the age-standardized burden rates of depressive disorders in China in 1990 and 2019 and the estimated annual percentage changes from 1990 to 2019

Characteristics	1990		2019		1990–2019
	Cases No.×10^6^ (95% UI)	ASR per 1000 No. (95% UI)	Cases No.×10^6^ (95% UI)	ASR per 1000 No. (95% UI)	AAPC in ASR No. (95% CI)
Incidence					
Overall					
*Both*	31.30 (27.14, 35.71)	26.48 (23.35, 29.91)	41.01 (36.46, 46.16)	23.01 (20.51, 25.71)	-0.82 (-1.00, -0.65)
*Male*	10.95 (9.52, 12.44)	18.31 (16.17, 20.62)	15.00 (13.36, 16.87)	16.89 (15.05, 18.86)	-0.84 (-1.03, -0.65)
*Female*	20.36 (17.62, 23.29)	35.00 (30.65, 39.56)	26.01 (23.18, 29.21)	29.22 (25.98, 32.71)	-0.84 (-1.04, -0.64)
MDD					
* Both*	28.95 (24.86, 33.30)	24.45 (21.35, 27.75)	37.51 (33.05, 42.36)	21.02 (18.55, 23.68)	-0.89 (-1.08, -0.70)
* Male*	9.95 (8.55, 11.43)	16.64 (14.54, 18.84)	13.55 (11.93, 15.39)	15.26 (13.47, 17.21)	-0.91 (-1.12, -0.70)
* Female*	19.00 (16.32, 21.84)	32.60 (28.42, 37.07)	23.96 (21.15, 27.05)	26.85 (23.71, 30.23)	-0.90 (-1.12, -0.68)
Dysthymia					
* Both*	2.35 (1.92, 2.84)	2.03 (1.66, 2.46)	3.49 (2.84, 4.26)	1.99 (1.65, 2.40)	-0.07 (-0.09, -0.05)
* Male*	1.00 (0.82, 1.21)	1.67 (1.38, 2.03)	1.45 (1.16, 1.78)	1.63 (1.33, 1.95)	-0.10 (-0.16, -0.05)
* Female*	1.35 (1.1, 1.64)	2.40 (1.94, 2.91)	2.05 (1.67, 2.50)	2.37 (1.96, 2.86)	-0.06 (-0.07, -0.05)
Prevalence					
Overall					
* Both*	34.14 (30.64, 37.99)	29.95 (27.03, 33.22)	50.06 (45.05, 55.58)	27.24 (24.6, 30.01)	-0.53 (-0.61, -0.45)
* Male*	12.77 (11.43, 14.26)	22.06 (19.82, 24.53)	18.99 (17.00, 21.2)	20.65 (18.63, 22.97)	-0.55 (-0.66, -0.44)
* Female*	21.37 (19.21, 23.83)	38.21 (34.54, 42.26)	31.07 (27.87, 34.5)	33.91 (30.57, 37.46)	-0.54 (-0.64, -0.44)
MDD					
* Both*	19.48 (16.79, 22.34)	16.49 (14.46, 18.62)	25.34 (22.41, 28.7)	14.13 (12.56, 15.93)	-0.90 (-1.09, -0.71)
* Male*	6.69 (5.76, 7.70)	11.22 (9.84, 12.70)	9.14 (8.08, 10.37)	10.25 (9.08, 11.57)	-0.93 (-1.14, -0.72)
* Female*	12.79 (11.05, 14.68)	21.99 (19.32, 24.85)	16.19 (14.31, 18.30)	18.04 (16.01, 20.32)	-0.92 (-1.13, -0.70)
Dysthymia					
* Both*	15.28 (12.92, 18.15)	14.02 (11.83, 16.69)	25.70 (21.61, 30.51)	13.61 (11.49, 16.00)	-0.11 (-0.18, -0.05)
* Male*	6.24 (5.21, 7.43)	11.12 (9.35, 13.22)	10.11 (8.45, 12.00)	10.68 (9.01, 12.56)	-0.16 (-0.26, -0.07)
* Female*	9.04 (7.62, 10.72)	17.06 (14.38, 20.34)	15.59 (13.03, 18.66)	16.59 (14.05, 19.67)	-0.10 (-0.15, -0.06)
DALYs					
Overall					
* Both*	5.49 (3.83, 7.54)	4.71 (3.31, 6.43)	7.56 (5.31, 10.43)	4.17 (2.93, 5.74)	-0.69 (-0.81, -0.57)
* Male*	2.00 (1.39, 2.75)	3.39 (2.37, 4.65)	2.84 (1.97, 3.91)	3.13 (2.19, 4.29)	-0.71 (-0.86, -0.57)
* Female*	3.49 (2.44, 4.79)	6.09 (4.30, 8.30)	4.72 (3.31, 6.47)	5.23 (3.65, 7.17)	-0.70 (-0.84, -0.56)
MDD					
* Both*	4.01 (2.74, 5.59)	3.36 (2.32, 4.64)	5.09 (3.51, 7.00)	2.85 (1.97, 3.93)	-0.94 (-1.12, -0.75)
* Male*	1.39 (0.95, 1.95)	2.31 (1.59, 3.18)	1.86 (1.27, 2.57)	2.09 (1.44, 2.88)	-0.96 (-1.17, -0.75)
* Female*	2.62 (1.79, 3.64)	4.46 (3.08, 6.18)	3.23 (2.23, 4.43)	3.63 (2.51, 4.99)	-0.95 (-1.16, -0.73)
Dysthymia					
* Both*	1.48 (0.96, 2.16)	1.35 (0.87, 1.96)	2.47 (1.60, 3.6)	1.32 (0.84, 1.91)	-0.10 (-0.16, -0.03)
* Male*	0.61 (0.39, 0.90)	1.08 (0.70, 1.60)	0.98 (0.63, 1.43)	1.04 (0.67, 1.52)	-0.16 (-0.26, -0.06)
* Female*	0.87 (0.57, 1.27)	1.63 (1.06, 2.38)	1.49 (0.96, 2.19)	1.60 (1.04, 2.34)	-0.09 (-0.13, -0.04)

ASR, age–standardized rate; UI: uncertainty interval; CI: confidence interval; DALYs: disability-adjusted life-years; AAPC: average annual percentage change

### The overall age-standardized estimates suggest that the burden of dysthymia remained relatively stable over the last three decades, while the burden of MDD decreased significantly in China

In terms of change from 1990 to 2019, the raw incidence, prevalence and DALYs of MDD and dysthymia in China all increased substantially (Table 1; Fig. 1). Notably, the raw number of prevalent cases of dysthymia [22.15 (95% UI 18.79, 26.35) million] surpassed MDD [21.86 (95% UI: 19.62, 24.25) million] for the first time since 2006, and increased in parallel with MDD in recent years (Fig. 1B). The ASIR of MDD decreased with an AAPC of -0.89 (95% CI: -1.08, -0.70), while the changes in the ASIR of dysthymia were negligible (Table 1; Fig. 1A). Both ASPR and ASDR of MDD showed a decrease with an AAPC of -0.90 (95% CI: -1.09, -0.71) and − 0.94 (95% CI: -1.12, -0.75), respectively. Similarly, the changes of ASPR and ASDR for dysthymia were almost negligible (Table 1; Fig. 1).

### Dysthymia remained relatively stable across most age groups, while the incidence of MDD tended to decrease in younger age groups and increase in older age groups

**Fig. 3:**
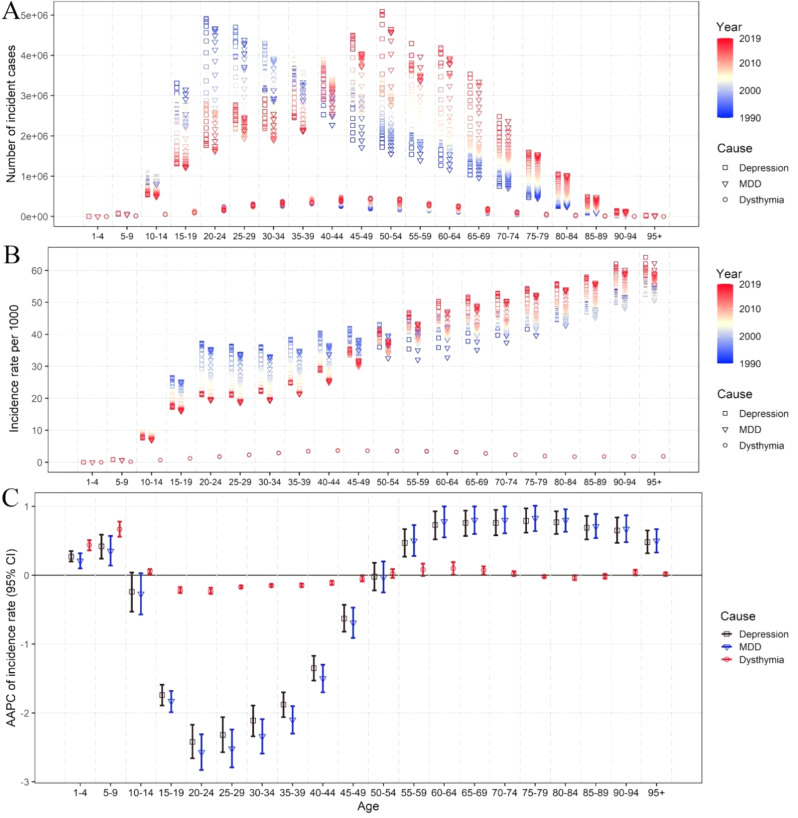
The incidence, incidence rate and AAPC of incidence rate of depressive disorders in China by categories and age. The numbers of incidence (**A**), rates of incidence (**B**), and AAPC of incidence rates (**C**) of depressive disorders by categories and ages in 1990–2019. AAPC, average annual percentage change

Over the past three decades, there has been a trend towards a decrease in the raw incidence of MDD in the 10-39-year-old age group, and an increase in the 45-94-year-old age group. Additionally, there has been a decrease in the AAPC of incidence rate in the 10–49 age group, but an increase in the 50–94 age group. On the other hand, dysthymia remained relatively stable in most age groups (Fig. 3A-C). The raw prevalence of MDD decreased between the ages of 10–39, and increased between the ages of 40–95. There was a decrease in the AAPC of prevalence rate in the 15–54 age group and an increase in the 55–95 age group, while dysthymia remained largely stable (Supporting Information: Fig S1). Similar results were observed for MDD and dysthymia DALYs (Supporting Information: Fig S2). In 2019, the rate of DALYs was increasing with aging for MDD, while ages of 60–64 have the top DALYs rate for dysthymia (Supporting Information: Fig 1S3). Overall, it appears that both the peak incidence and prevalence of MDD are gradually aging, while dysthymia remained largely stable during the past thirty years.

### The burden of depressive disorders attributable to intimate partner violence was found to be more pronounced in women, particularly in older age groups

In 2019, the raw incidence of depressive disorders was highest in both sexes, with 26.01 million cases (95% CI: 23.18, 29.21) in women and 15.00 million cases (95% UI: 13.36, 16.87) in men (Table 1). Similarly, the prevalence of depressive disorders was higher in women (31.07 million cases; 95% UI: 27.87, 34.50) than in men (18.99 million cases; 95% UI: 17.00, 21.20). The burden of depressive disorders, as measured by DALYs, was also higher in women, with 4.72 million DALYs (95% UI: 3.31, 6.47), compared to 2.84 million DALYs (95% UI: 1.97, 3.91) in men (Table 1).

**Fig. 4:**
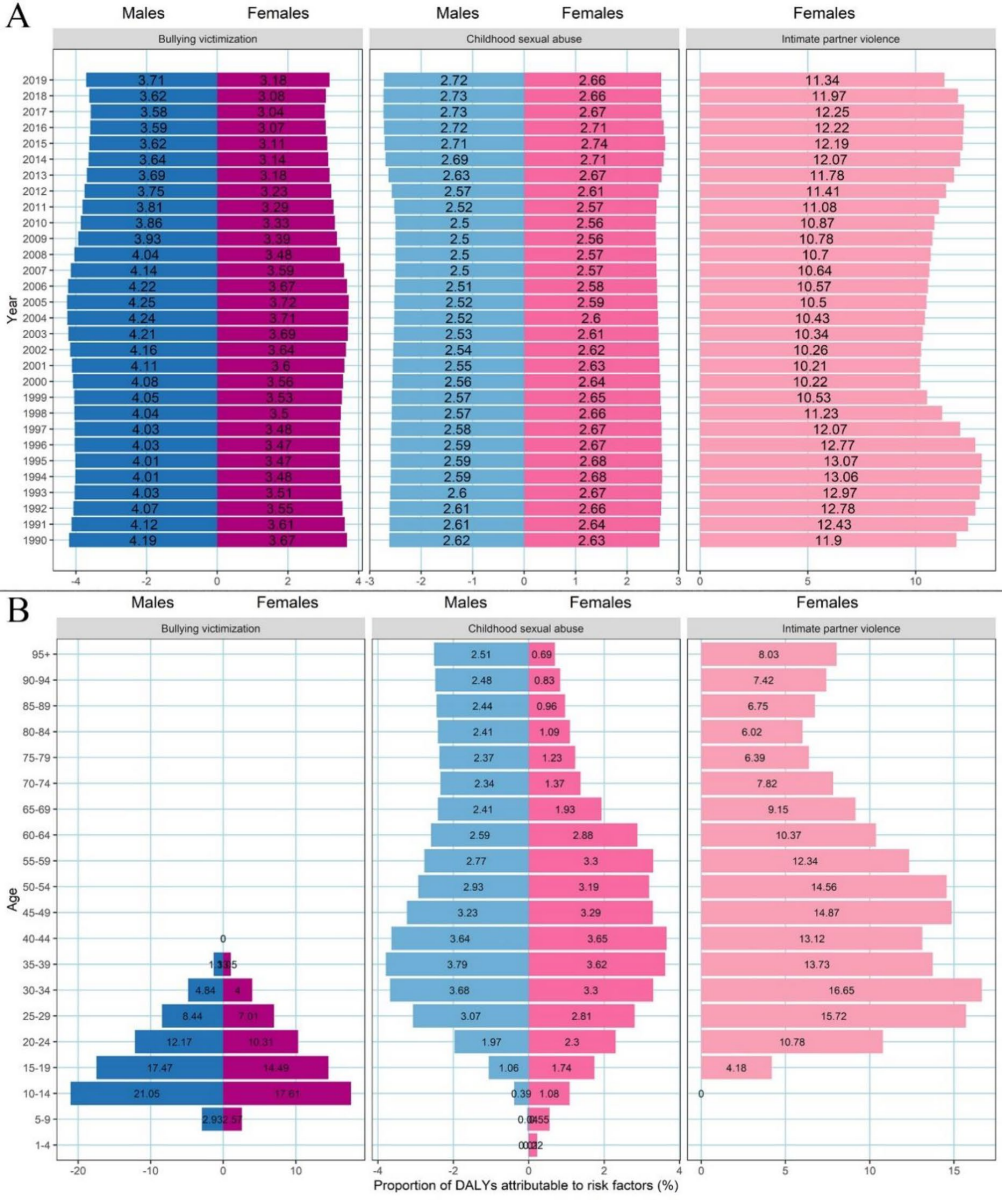
Proportions of DALYs attributable to risk factors in China by calendar year and age group. Proportions of DALYs attributable to risk factors by sex in China from 1990 to 2019 (**A**). Proportions of DALYs attributable to risk factors by age and sex in 2019 in China (**B**)

The DALYs attributable to intimate partner violence were the highest among all risk factors for depressive disorders in China in 2019, at 11.34% (95% UI: 0.07%, 29.51%). While the population attributable fractions of bullying victimization and childhood sexual abuse remained relatively stable over time for both sexes, the population attributable fraction of intimate partner violence was higher between 1990 and 1998 and then showed a decline between 1999 and 2010 before rising again in women from 2011 (Fig. 4A). The proportions of DALYs attributable to risk factors were similar in both sexes, with bullying victimization contributing to the burden of depressive disorders in men and women aged 5–39 years (concentrated in the 10–24 age group), while childhood sexual violence contributed to the burden across all age groups (Fig. 4B). The burden of depression attributable to intimate partner violence was concentrated in women aged 20–59 years. Over the past three decades, the burden of depressive disorders attributable to childhood sexual abuse decreased in the 15–44 age group but increased in the 50–84 age group. The proportion of DALYs attributable to intimate partner violence in women aged 20–54 years decreased over time (Supporting Information: Fig S4).

### The age-standardized burden of depressive disorders in China is expected to decline between 2020 and 2030, with a slower decline of dysthymia compared to MDD

**Fig. 5:**
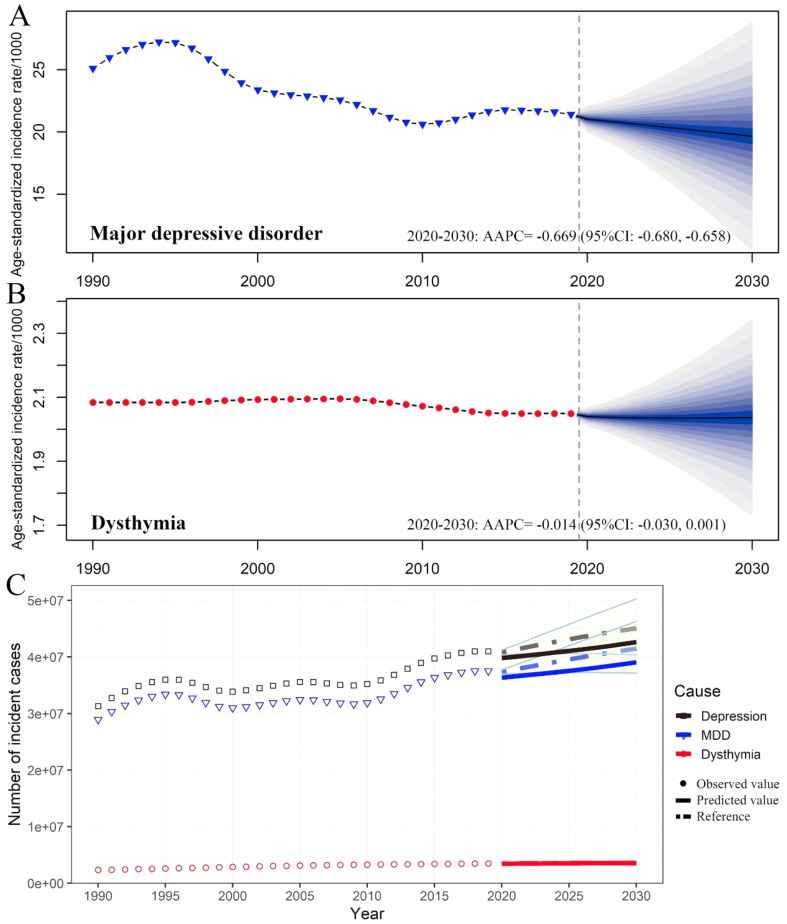
Trends of the age-standardized incidence rate and incidence of depressive disorders in China by categories. Trends of age-standardized incidence rate of MDD (**A**) and dysthymia (**B**) in China: observed (solid lines) and predicted rates (dashed lines). Trends MDD and dysthymia in the number of incidence cases (**C**): Solid lines and dash lines represent the observed and the predicted number of incident cases of depressive disorders; shading represents a 1% decrease and increases interval based on the 2019 rate

Based on the GBD data of depressive disorders in China from 1990 to 2019, it is predicted that the burden of depressive disorders will continue to increase between 2020 and 2030, with a notable exacerbation of dysthymia compared to MDD. The raw number of incidence cases of depressive disorders is expected to increase to 42.63 million, with MDD and dysthymia cases increasing to 39.07 million and 3.55 million, respectively (a 4.16% and 1.72% increase from 2019) (Fig. 5C).

**Fig. 6:**
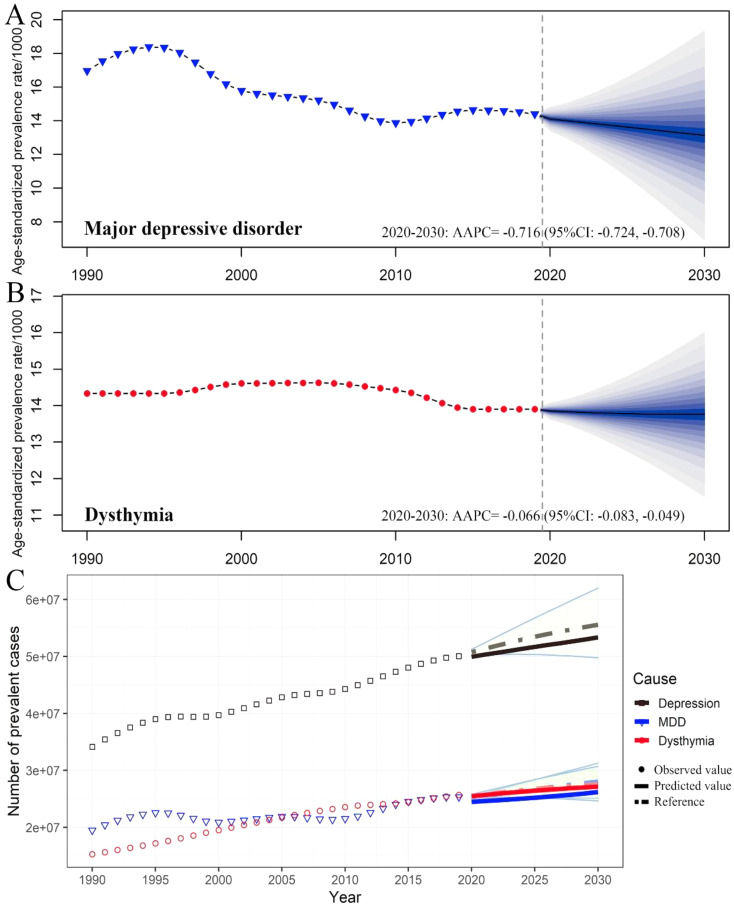
Trends of the age-standardized prevalence rate and prevalence of depressive disorders in China by categories. Trends of the age-standardized incidence rate of MDD (**A**) and dysthymia (**B**): observed (solid lines) and predicted rates (dashed lines). Trends of prevalence cases of depressive disorder by categories (**C**): Solid lines and dash lines represent the observed and the predicted number of prevalent cases of depressive disorders; shading represents a 1% decrease and increases interval based on the 2019 rate

In addition, the prevalence of depressive disorders is expected to increase to 53.35 million, with MDD and dysthymia cases increasing to 26.20 million and 27.15 million, respectively (a 3.39% and 5.64% increase from 2019) (Fig. 6C).

**Fig. 7:**
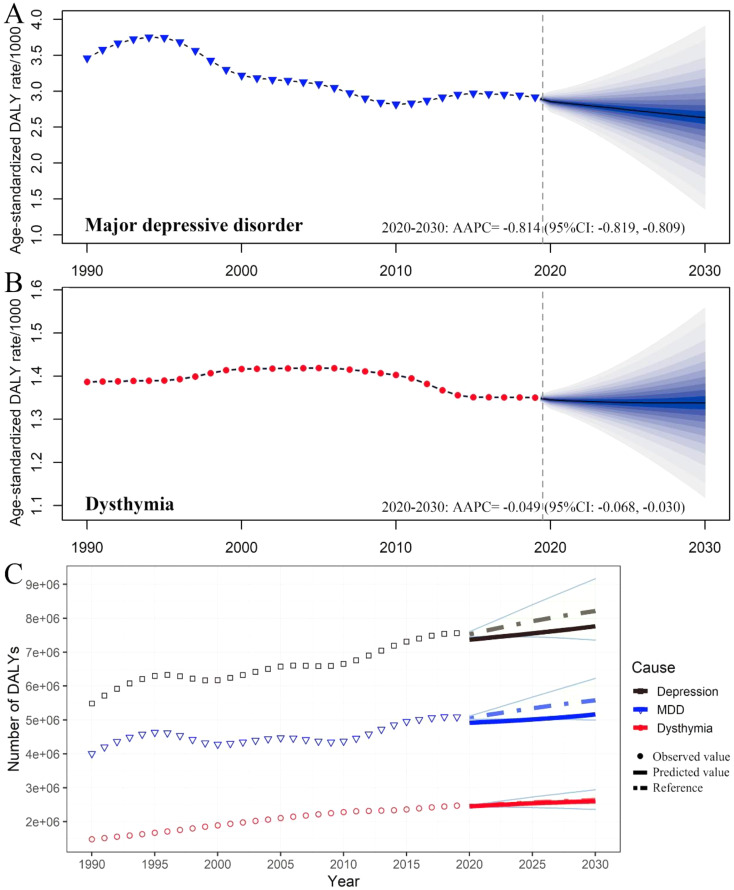
Trends of the age-standardized of DALYs rates and DALYs of depressive disorders in China by categories. Trends of the age-standardized of DALYs rates of MDD (**A**) and dysthymia (**B**) in China: observed (solid lines) and predicted rates (dashed lines). Trends of DALYs of depressive disorder by categories (**C**): Solid lines and dash lines represent the observed and the predicted number of prevalent cases of depressive disorders; shading represents a 1% decrease and increases interval based on the 2019 rate

Moreover, the number of DALYs due to depressive disorders is expected to increase to 7.77 million, with MDD and dysthymia cases accounting for 5.16 million and 2.6 million DALYs, respectively (a 1.38% and 5.26% increase from 2019) (Fig. 7C). Notably, dysthymia is expected to increase at a faster rate than MDD in both raw prevalence and DALYs. When age is standardized, MDD is projected to show a downward trend, with the AAPC of ASIR, ASPR, and DALYs being − 0.669 (95% CI: -0.680, -0.658) (Fig. 5A), -0.716 (95% CI: -0.680, -0.658) (Fig. 6A), and − 0.814 (95% CI: -0.819, -0.809) (Fig. 7A), respectively. Meanwhile, the trend in dysthymia burden is expected to remain largely stable (Figs. 5B, 6B and 7B) when age is standardized.

## Discussion

This study used the GBD 2019 study to compare and analyze the disease burden of MDD and dysthymia in China, and further predict the incidence, prevalence, and DALYs in the next 11 years. The burden of depressive disorders in China is heavy due to the huge population size, and it is expected that the burden will continue to increase from 2020 to 2030. In particular, dysthymia, once underestimated by many as a personality style rather than a mood disorder because of the low-grade persistent symptoms [8], has shown a significant increase in the numbers of incidence, prevalence, and DALYs over the past three decades, and will continue to grow significantly from in the next 11 years. Therefore, the government, experts and even the public need to pay more attention.

Recent research has challenged the previous notion that dysthymia is only a mild condition [8]. Studies have shown that first-degree relatives of patients with dysthymia have a higher rate of major depressive disorder than relatives of healthy controls [22]. Dysthymia is also associated with an increased risk of suicide [23], and almost all patients with dysthymia will eventually have a major depressive episode [24]. However, dysthymia is often overlooked due to its mild symptoms or short-term, narrow assessment methods [8, 25]. Moreover, individuals with persistent depressive disorder may ignore dysthymia until it is exacerbated in the form of a major depressive episode [8]. The current study revealed a rise in the absolute number of DALYs associated with MDD, while the age-standardized rate of DALYs for MDD also showed an upward trend, which could potentially be attributed to the rapid growth of the senior population in China. In fact, raw rates are determined by considering the entire population being studied, taking into account the age distribution of the population, while standardised rates, rely on a fixed age structure and are not influenced by shifts in the population’s age distribution. Based on the findings of the seventh national census conducted in 2020, those aged 60 and above constituted 18.7% of China’s overall population, amounting to 264 million people [26]. Furthermore, there has been a significant surge in the prevalence of depression among the senior population in China. The senior population may have greater risk factors that lead to more severe depressive symptoms or episodes, which are subsequently diagnosed as MDD. However, the disparity between MDD and dysthymia could account for the observed lesser change in dysthymia. Dysthymia can present itself in subtle and persistent ways, making it challenging to diagnose. It is particularly difficult to identify in primary care settings until it worsens and overlaps with a major depressive disorder episode [27].

Notably, although the incidence of dysthymia has been much lower than MDD over the past three decades, its prevalence has unexpectedly been higher than MDD since 2006 and will continue to grow faster than MDD. Possible reasons include the fact that current dimensional classification systems focus more on cross-sectional symptomatology and less on longitudinal aspects, such as symptom development and disease course. This tendency induces clinicians to focus more on emergencies and overlook current dysthymia [28]. With increasing exposure time, clinicians gradually revise their clinical diagnosis. The chronic nature of dysthymia also requires longer and more structured treatments to address the high risk of recurrence [29]. However, only nearly one-third of patients with persistent depression, such as dysthymia, receive adequate doses and duration of medication [30], even though existing pharmacotherapy interventions may be more effective in chronic dysthymia than in non-chronic MDD [31].Patients with chronic dysthymic symptoms also have lower rates of spontaneous remission [32]. Taken together, these factors result in a much lower incidence but higher prevalence of dysthymia compared to MDD.

In line with previous research by Ren et al., the current study found an increase in incident cases, prevalent cases, and DALYs of depressive disorders in China during the study period [2]. However, this study also identified a rise in age-specific incidence, prevalence, and DALY rates among individuals aged 50 years and above, which was not reported in Ren et al.‘s study. As China is transitioning from a mildly aging country to a moderately aging one, its elderly population may surpass 300 million by 2025, necessitating increased attention from the government and society towards this demographic [33]. A previous study indicated that individuals aged 60 or older who underwent depressive symptom screening had a high prevalence of depressive symptoms at 15.94%, underscoring depression as a complicated public health issue among older adults in China [34]. Similarly, prior research has demonstrated that older adults have a higher incidence and prevalence of depressive disorders, and the risk of depression in middle-aged and elderly individuals in China is also elevated at 25.17%, which rises with increasing age [35]. Elderly individuals with more than two chronic diseases are at a higher risk of developing depression [36]. The socio-cultural environment in China can lead to the elderly perceiving themselves as a burden to their families and society, which may gradually lead to depression [37]. Furthermore, there is often a long-term depression around retirement age, and compared to retiring or being inactive, working in the long-term is associated with lower depressive symptoms [38]. With the impact of the “one-child policy” and the rapid aging of the population, the burden of disease is expected to increase in the future.

The results of present study showed that intimate partner violence was the most significant contributor to the burden of depression in China, followed by bullying victimization and child sexual violence. In recent years, the level of intimate partner violence has gradually increased in China, which can lead to victims’ feelings of fear, helplessness, powerlessness, and isolation [39]. Meta-analyses have indicated that the prevalence of depression is high among women who have experienced psychological violence (65.8%), physical violence (69.5%), and sexual violence (75.8%) [40]. The study also confirmed that the tendency of young women to suffer from intimate partner violence has decreased in the past decades but has increased in recent years. In China, the DALYs rate per 10,000 elderly women suffering from intimate partner violence has unexpectedly increased in recent years. This could be due to the social status of women and increasing economic stress, which predisposes them to anger, frustration, and manifestations of violence [41, 42]. These economic pressures, and the resulting changes in perception, ultimately lead to a higher risk of intimate partner violence. Furthermore, bullying victimization, which commonly occurs among school-aged children, is a salient stressor that leads to deficits in emotion regulation across the lifespan [43]. There is strong evidence that bullying victimization is associated with suicidal ideation and attempts and that depression is a major undesirable outcome. Depression was found to be a moderator between bullying and suicidality [44–46]. In addition, the estimated prevalence of child sexual violence among males and females in China was 9.1% and 8.9%, respectively. The prevalence of male child sexual violence in China is higher than the global prevalence (7.9%) [47, 48]. Childhood sexual violence has negative effects on victims’ physical and mental health and is associated with an increased risk of depression [49]. Although the risk of child sexual violence has remained stable from 1990 to 2019, the problem still needs to be taken seriously. Early intervention to identify and support victims of life trauma could prevent the development of nasty conditions.

According to the WHO, depressive disorders are one of the leading causes of disability worldwide and contribute to the global burden of disease, accounting for about 46.9 million DALYs in 2019 [14]. However, in China, ASIR, ASPR, and ASDR are lower than in numerous developed countries. It is possible that this data is underestimated due to stigma and/or lack of mental health knowledge. Only 9.5% of people with depressive disorders used mental health services [10], much lower than in the United States (57.3% for MDD) [50] and other high-income countries [51], which confirms this speculation. Moreover, the uneven distribution of healthcare resources in China, especially the shortage of mental health services and inadequate training of mental health workers in western rural areas, has led to low diagnosis rates of depressive disorders [52]. Patients with depression often prefer to visit local tertiary or secondary general hospitals first, rather than a psychiatric specialist. Additionally, the Chinese are more likely to seek help from physicians in traditional Chinese medicine, resulting in some depressed patients being diagnosed with “mental disorders” instead of depressive disorders [10]. Furthermore, among depressed patients who first seek treatment in psychiatric hospitals, the number of those who worsen due to poor treatment or excessive medical expenses is staggering, leading to a waste of medical resources [53]. Therefore, the media, schools, and communities should strengthen mental health education and popularize relevant knowledge for the public in China. Moreover, psychological consulting and therapy should be a conventional procedure added to the therapy process of depressive disorders. Especially since the aging population is growing fast, the higher incidence rates of depression among the elderly remind us of the need to pay special attention to this group. It is meaningful to create a friendly social environment for the elderly and establish a positive attitude towards aging in the whole society [54]. Therefore, it is essential that the allocation of medical resources in China be based on the demographic characteristics of the disease burden.

## Limitations

While this study sheds light on the burden of depression in China, there are some limitations to consider. Firstly, the estimates provided in GBD 2019 are based on mathematical modeling, and further research is needed to reflect a more realistic burden of disease. Secondly, depression in China carries a significant social stigma that can lead to underdiagnosis and may skew the estimated burden of depression in the country. Thirdly, this study only analyzed the national disease burden of depression and did not investigate the prevalence, incidence, DALYs, and risk factors of depression in different provincial or economic regions of China. Future research should focus on addressing these limitations to provide a more comprehensive picture of depression in China.

## Conclusion

In conclusion, it is evident that the burden of depressive disorders in China has been increasing over the past 30 years due to various factors such as a large population, aging, economic pressures, and poor treatment, and it is expected to continue increasing in the future. Dysthymia, which was previously overlooked, is now receiving more attention due to its similar disability and clinical severity as MDD, and the detection rate of dysthymia is gradually increasing, leading to a shift in the subtypes of depressive disorders. MDD has seen a decrease in all age-standardized indicators, and the peak population of patients tends to be older, while dysthymia has remained relatively stable. It is crucial to establish targeted prevention and treatment strategies based on the existing population structure and risk factors. These strategies should focus on early identification and treatment of dysthymia, mental health education, attention to population aging, and early intervention for individuals suffering from life trauma. By implementing these multiple strategies, the burden of depressive disorders can be reduced in China.

### Electronic supplementary material

Below is the link to the electronic supplementary material.

Supplementary Material 1

## Data Availability

The datasets generated and/or analysed during the current study are available in the online database (http://ghdx.healthdata.org/gbd-results-tool*)*
