# Supplementary material for: Notable dysthymia: evolving trends of major depressive disorders and dysthymia in China from 1990 to 2019, and projections until 2030
Source: BMC Public Health. 2024 Jun 13;24:1585. doi: 10.1186/s12889-024-18943-7 (PMC11170895; doi:10.1186/s12889-024-18943-7)
Supplement: Supplementary file 1 — Supplementary Material 1 [file 12889_2024_18943_MOESM1_ESM.docx]

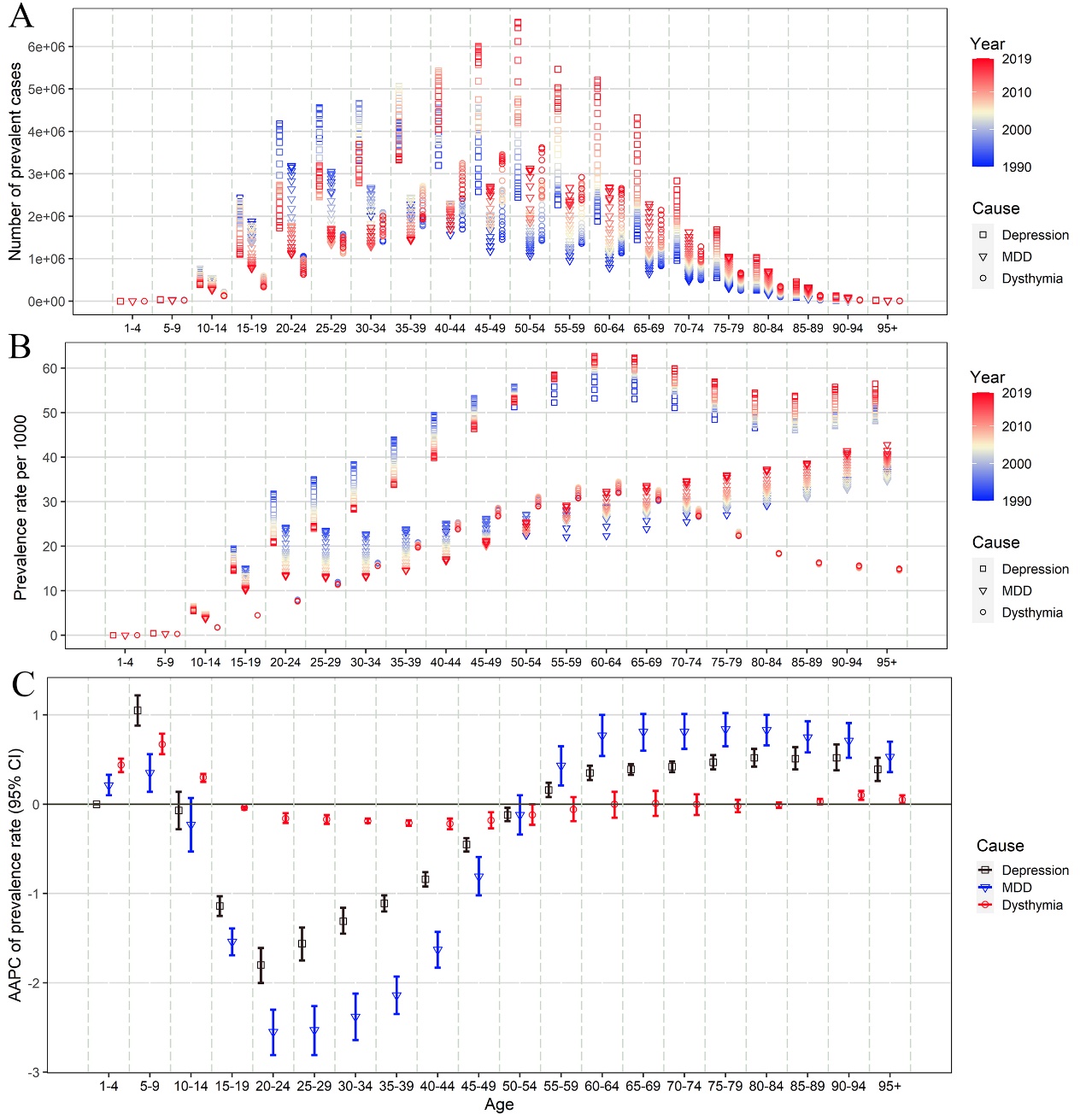


**Fig S1. The prevalence, prevalence rate and AAPC of prevalence rate of depressive disorders in China by categories and age.** The numbers of prevalence (A), rates of prevalence (B), and AAPC of prevalence rates (C) of depression by categories and ages in 1990-2019. AAPC, average annual percentage change.

**
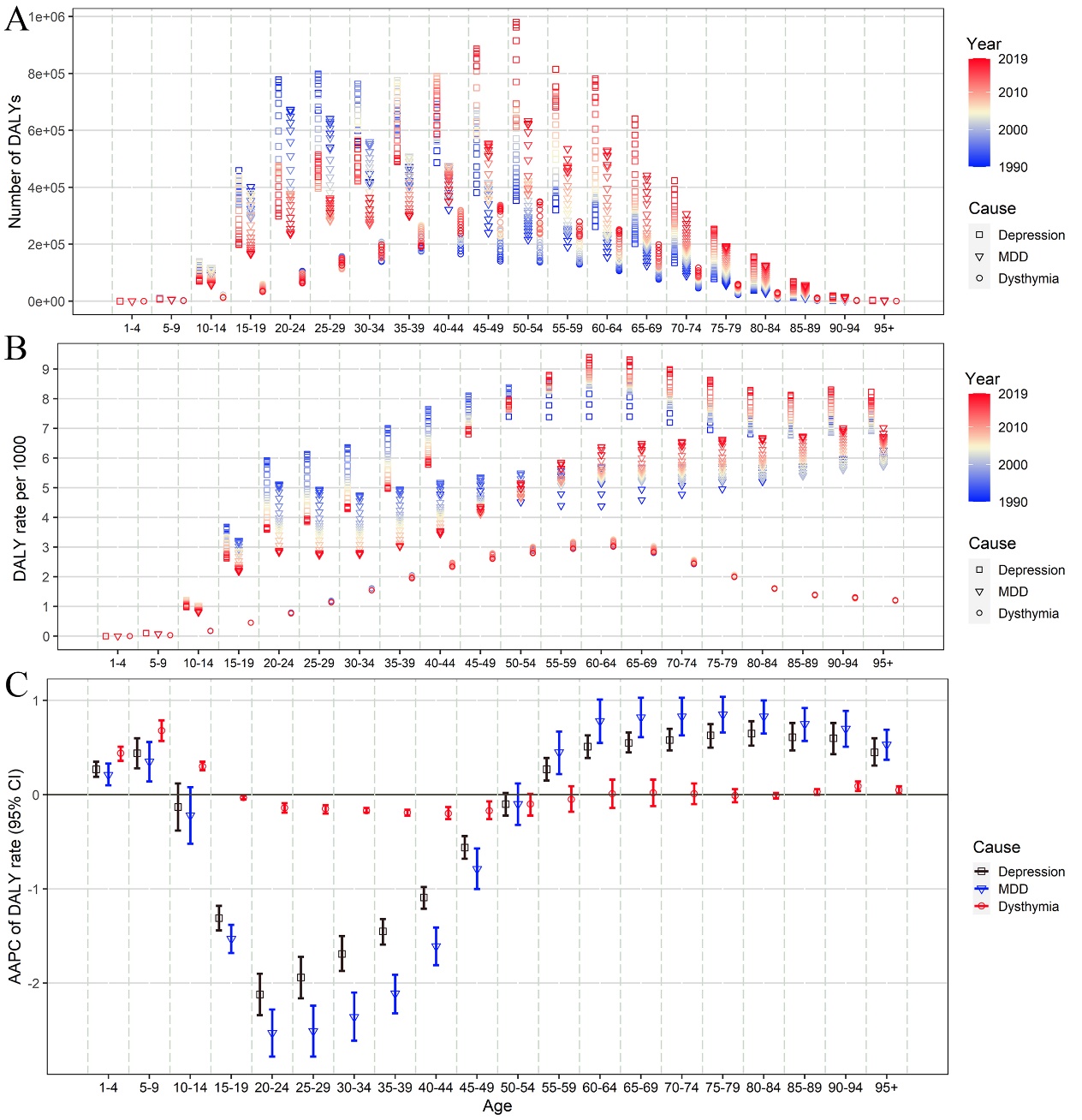
**

**Fig S2. The DALYs, DALYs rate and AAPC of DALYs rate of depressive disorders in China by categories and age.** The numbers of DALYs (A), rates of DALYs (B), and AAPC of DALYs rates (C) of depressive disorders by categories and ages in 1990-2019. AAPC, average annual percentage change. DALYs, disability-adjusted life-year.

**
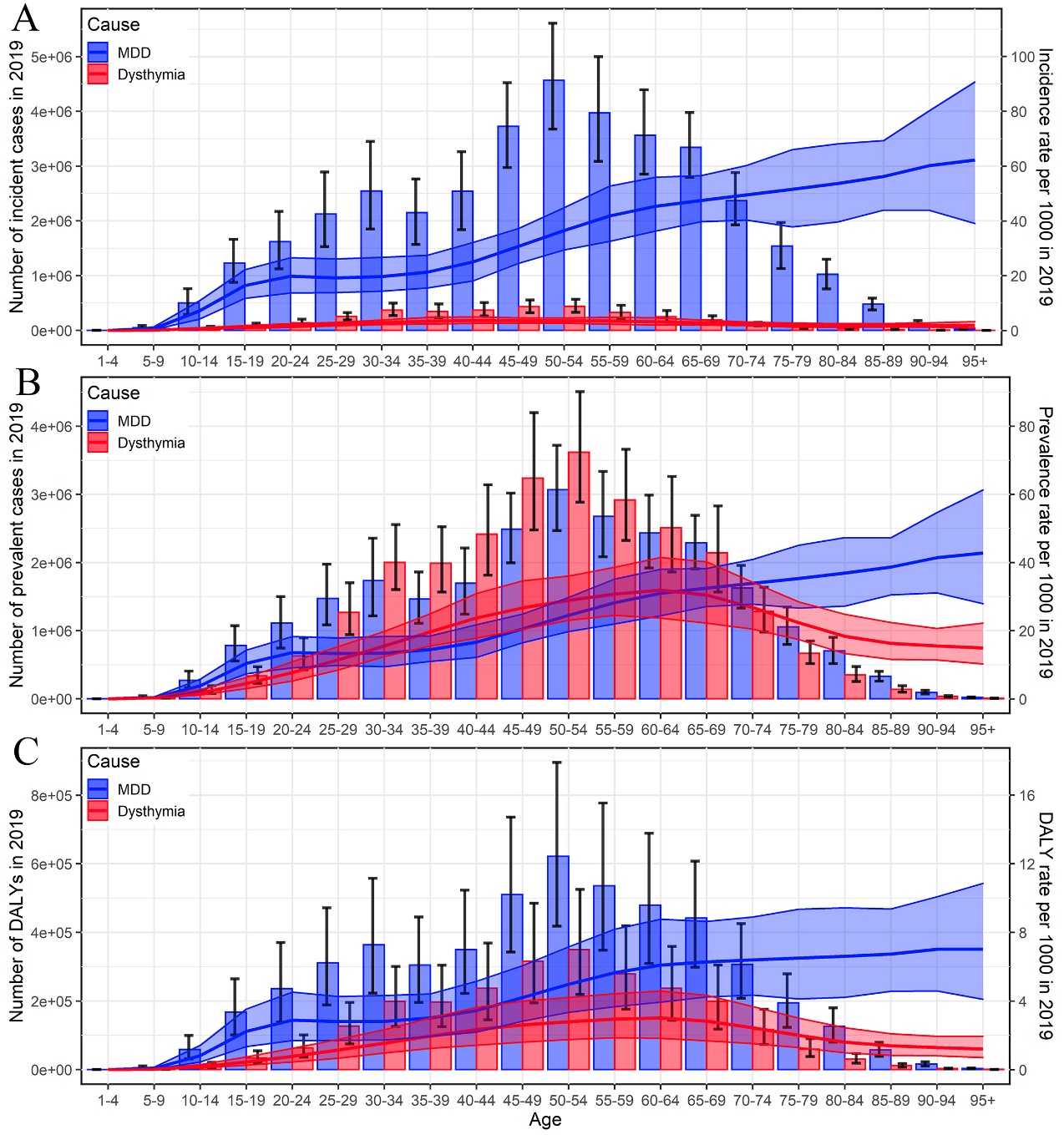
**

**Fig S3. The incidence, prevalence, and DALYs of depressive disorders in China by categories and age in 2019.** The numbers and rates of incidence (A), prevalence (B), and DALYs (C) of depression by categories and ages in 2019. DALYs, disability-adjusted life-year.


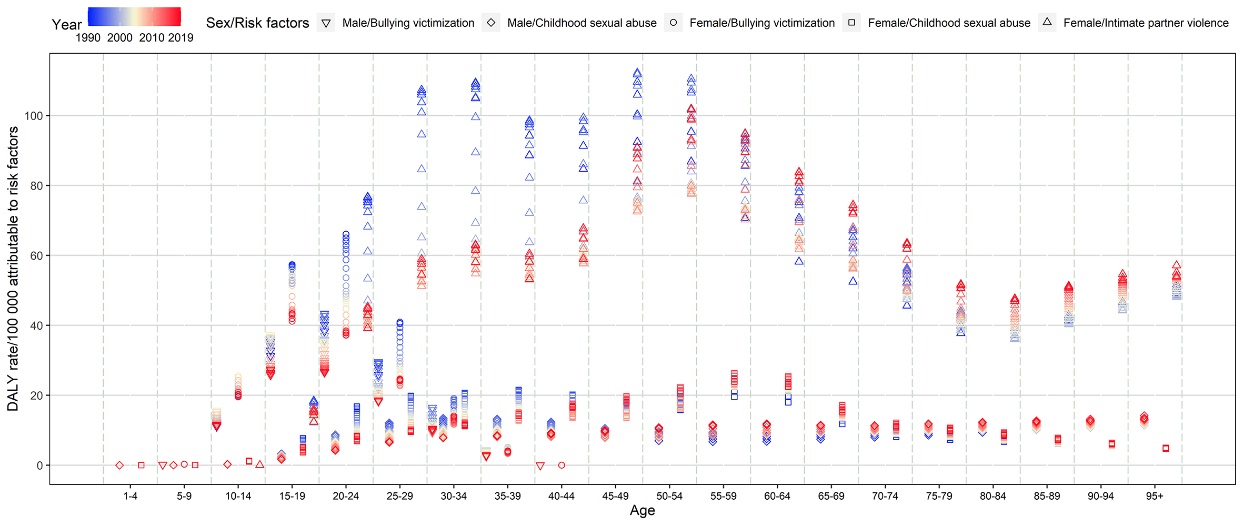


**Fig S4. Proportions of DALYs attributable to risk factors by sex in 2019 in China.** DALYs, disability-adjusted life-year.
